# Headway in multiple sclerosis therapy: the case for platinum analogues

**DOI:** 10.1097/MS9.0000000000003536

**Published:** 2025-07-09

**Authors:** Zain Ali Nadeem, Umar Akram, Aalaa Saleh, Aimen Nadeem

**Affiliations:** a Department of Medicine, Allama Iqbal Medical College, Lahore, Pakistan; b Faculty of Medicine, Lebanese University, Beirut, Lebanon; c Department of Medicine, King Edward Medical University, Lahore, Pakistan

**Keywords:** carboplatin, cisplatin, multiple sclerosis, platinum analogues

## Abstract

Multiple sclerosis is a chronic neuroinflammatory disease associated with significant morbidity. Currently available pharmacotherapeutics are associated with several adverse effects. This letter discusses the potential of carboplatin, a platinum analog, which has recently shown promising results. We delve into the mechanisms of action and review the literature for potential adverse effects of carboplatin therapy.

*To the Editor*,

Multiple sclerosis (MS) is a widely prevalent neurodegenerative autoimmune condition that greatly deteriorates quality of life. The central nervous system (CNS) components – myelin, oligodendrocytes, and neurons – are damaged by immune cells; both T cells and B cells are implicated in the pathology. MS remains incurable, although disease-modifying therapy is directed toward delaying progression and lessening relapses[1]. However, recent investigations exploring the use of carboplatin, a platinum-based chemotherapeutic agent, provide encouraging results and suggest its potential as a novel therapeutic approach for MS. Here, we discuss the potential mechanisms of action of carboplatin, comparison with existing therapies and possible uses in combination treatment regimens, its adverse effects, and future clinical and research implications. This paper is compliant with the Transparency In The reporting of Artificial Intelligence (TITAN) guideline, with no use of artificial intelligence at any point^2^.

In a study on an animal model of experimental autoimmune encephalomyelitis (EAE), the authors explored the effect of carboplatin on disease pathophysiology,^3^. The authors reported that carboplatin delays the onset of clinical symptoms in mice, reduces the severity of the disease, decreases spinal cord inflammation and demyelination, and is effective even after disease onset. Additionally, carboplatin decreases the number of autoreactive T cells in the spleen and lymph nodes, particularly Th1 and Th17 cells, which are major contributors to CNS inflammation in MS. Proteomic analysis and *in vitro* experiments confirmed that carboplatin inhibits T cell proliferation and induces apoptosis of activated T cells and myelin oligodendrocyte glycoprotein-specific T cells. These results suggest that carboplatin protects against the development and progression of EAE and, by extension, might earn a right to exploration as a novel therapeutic drug for MS. Similar mechanisms of action have been proposed for interferon-β and glutiramer acetate. Interferon-β shows limited efficacy in late stages of the disease and may exacerbate neuromyelitis optica due to its activation of CD8+ cytotoxic T cells, while glatiramer acetate has a more specific anti-inflammatory effect. Carboplatin could augment these effects in combination regimens,^[4]^.

EAE is the common experimental model for MS to explore treatment approaches^[5]^. Most of the currently approved therapies for MS were either initially tested on EAE models or were later found to be effective in EAE. Promising outcomes in EAE models would warrant further consideration. Furthermore, carboplatin is a familiar territory. Much is already known about its mechanism of action and safety profile[5]. It is known for inducing apoptosis in cancer cell lines via multiple mechanisms^[6,7]^. Carboplatin is currently the treatment of choice for many cancers, such as ovarian cancer, lung cancer, and head and neck cancers, replacing cisplatin due to the lesser side effects of the former[5]. Additionally, mounting evidence in recent years advocates for the safety of carboplatin in pregnancy^[8,9]^.

However, the drug is not without pitfalls. Myelosuppression is a major dose-limiting adverse effect that poses many problems^[10]^. Another upsetting adverse outcome of platinum-based analogues seems to be Lhermitte’s phenomenon^[11]^. Considering Lhermitte’s phenomenon is a common impairment that many persons with MS already face, the capacity of carboplatin to possibly exacerbate the condition must be thoroughly examined. Furthermore, carboplatin has limited blood–brain barrier (BBB) permeability, and techniques such as temporary BBB disruption using an ultrasound machine are being explored to increase the penetration of the drug across the BBB^[12]^. To address the concern for accelerating MS progression, the simultaneous use of monoclonal antibodies such as ocrelizumab and alemtuzumab that deplete the lymphocytes responsible for MS can be explored^[13]^.

Nevertheless, carboplatin is a promising new therapeutic approach to MS. Further avid research could unlock new treatments for the disease, and the focus on carboplatin to discover its true potential could provide a much-needed breakthrough. Future research should involve clinical trials testing the efficacy of carboplatin in humans with MS in combination with different existing treatments to check any benefit with combination therapy. Techniques to increase the BBB-permeability of carboplatin should be explored, and the risks and benefits should be carefully assessed.

## Data Availability

No datasets were generated or analyzed during this study.
